# Scattering-type scanning near-field optical microscopy with low-repetition-rate pulsed light source through phase-domain sampling

**DOI:** 10.1038/ncomms13212

**Published:** 2016-10-17

**Authors:** Haomin Wang, Le Wang, Xiaoji G. Xu

**Affiliations:** 1 Department of Chemistry, Lehigh University, 6 E Packer Avenue, Bethlehem, Pennsylvania 18015, USA

## Abstract

Scattering-type scanning near-field optical microscopy (s-SNOM) allows spectroscopic imaging with spatial resolution below the diffraction limit. With suitable light sources, s-SNOM is instrumental in numerous discoveries at the nanoscale. So far, the light sources have been limited to continuous wave or high-repetition-rate pulsed lasers. Low-repetition-rate pulsed sources cannot be used, due to the limitation of the lock-in detection mechanism that is required for current s-SNOM techniques. Here, we report a near-field signal extraction method that enables low-repetition-rate pulsed light sources. The method correlates scattering signals from pulses with the mechanical phases of the oscillating s-SNOM probe to obtain near-field signal, by-passing the apparent restriction imposed by the Nyquist–Shannon sampling theorem on the repetition rate. The method shall enable s-SNOM with low-repetition-rate pulses with high-peak-powers, such as femtosecond laser amplifiers, to facilitate investigations of strong light–matter interactions and nonlinear processes at the nanoscale.

## Abstract (web summary)

Low repetition rate lasers are suitable for studying nonlinear optical phenomena, while near-field microscopy allows high spatial resolution for nanomaterial characterisation. Here, Wang *et al*. enable scattering-type near-field microscopy with low repetition rate lasers through phase-domain sampling.

## Graphical abstract

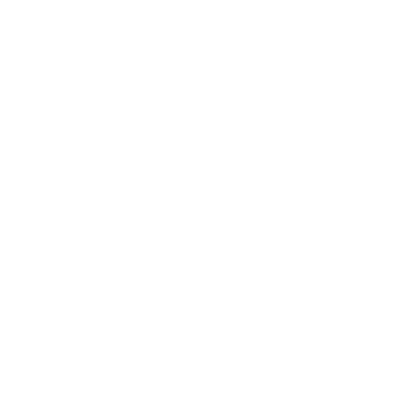

The spatial resolution of conventional far-field spectroscopy is bound by the far-field diffraction limit to about a half of the wavelength^1^,^2^. To overcome this limitation, scattering-type scanning near-field optical microscopy (s-SNOM) provides a generally applicable, label-free method for nanoscale surface characterizations^3^,^4^. A wide range of discoveries in the condensed phase materials, and two-dimensional materials have been facilitated by s-SNOM (refs 5, 6, 7, 8, 9, 10, 11, 12). In s-SNOM, an atomic force microscope (AFM) drives a sharp metallic or metal-coated tip in tapping mode or intermittent contact mode to scan over the sample of interest; an external light source is coupled to the tip apex and the scattered light from the tip with sample underneath is collected, then converted into electric signal by an optical detector. The signal is routed to a lock-in amplifier, where the non-fundamental harmonic demodulations can be extracted with the tip oscillation frequency as the reference frequency. The optical frequency of the external radiation is chosen to match the energy of a particular physical process, such as electronic, vibrational or polaritonic resonances. Various light sources have been used in s-SNOM, with frequencies ranging from visible light to terahertz^13^,^14^,^15^,^16^,^17^,^18^. The light source can be either coherent sources: such as gas lasers^19^, solid-state lasers, free electron lasers^20^ or incoherent sources, such as synchrotron radiation^21^,^22^ or a heated blackbody thermal source^23^. The wide availability of light sources further improves the applicability of s-SNOM techniques towards becoming a standard for high-spatial-resolution surface spectroscopic analysis.

However, not all light sources are suitable for s-SNOM. To extract near-field signal with lock-in detection, the light radiation has to be either continuous or pulsed at a much higher repetition rate than the mechanical oscillation frequency of the tip^24^,^25^,^26^. For example, the infrared radiation from the difference frequency generation from femtosecond Erbium fibre lasers has a repetition rate of about 40 or 80 MHz, whereas the AFM tip oscillates at frequencies of ∼300 kHz or lower. In lock-in detection, the reference frequency of the lock-in amplifier is set at a non-fundamental harmonic (≥2) of the tip oscillation frequency^27^. Signals from non-fundamental harmonic demodulation are present, because the short-range tip–sample near-field interactions generate anharmonic components in the scattering waveform, while the tip is purely harmonically oscillating above the sample. However, if the repetition rate of the pulsed laser is lower than the reference frequency, that is, tip oscillation frequency, the lock-in amplifier is unable to generate meaningful demodulation signals that manifest near-field interactions, because the near-field interaction is under-sampled in the time domain by the sparsely timed pulses that generate detectable signals. The Nyquist–Shannon sampling theorem states that for an effective sampling process, the sampling rate should be more than the critical sample rate, which is twice of the target frequency^28^. For the second-harmonic demodulation with a lock-in amplifier, proper sampling requires the pulse repetition rate to be at least four times of the tip oscillation frequency or six times for the third-harmonic demodulation. Also, for an s-SNOM apparatus coupled with low-repetition-rate pulsed lasers, the effective sampling rate of the lock-in detection is limited by the repetition rate of the laser. For a tip of 250 kHz oscillation frequency, the repetition rate of the pulsed laser has to be at least 1 MHz for the second-harmonic demodulation. At the lower limit of the critical sampling rate with lock-in detection, additional considerations have to be taken into account, such as synchronization of the detector sampling rate^25^ or tip oscillation frequency^29^ with the pulsed laser source to maintain the near-field contrast.

Low-repetition-rate pulsed laser systems are associated with desirable attributes. For example, the high-peak power of solid-state femtosecond amplifier is suitable for frequency conversions through nonlinear processes, such as the difference frequency generation of mid-infrared radiation^30^. A pulsed laser with high-repetition rate, however, is not efficient on nonlinear processes due to its low-peak power, given the same average power. For instance, the mid-infrared outputs in the fingerprint region are on order of a mere 1 mW from difference frequency generations from the solid-state Ti:sapphire oscillator or Erbium fibre lasers with 40, or 80 MHz repetition rates^16^,^31^,^32^. In comparison, the output of difference frequency generation from optical parametric amplifier pumped by a 250 kHz repetition rate Ti:sapphire regenerative amplifier system could exceed 10 mW even 20 years ago^33^. In addition, the high-peak power of femtosecond amplifier system of low-repetition rate is suitable for accessing strong light–matter interactions and nonlinear optical phenomena with s-SNOM, an area which remains largely under-explored and holds great promise for new discoveries^34^,^35^.

How could one operate s-SNOM with low-repetition rate pulsed light sources? In this article, we report a detection mechanism that works with a pulsed light source of repetition rates much lower than the tip oscillation frequency. The detection method allows reconstruction of tip–sample near-field responses, which provides the equivalent information to the current s-SNOM techniques with lock-in detection. This new detection technique will enable operations of s-SNOM with a range of pulsed laser systems.

## Results

### Principle of the method

**Figure 1:**
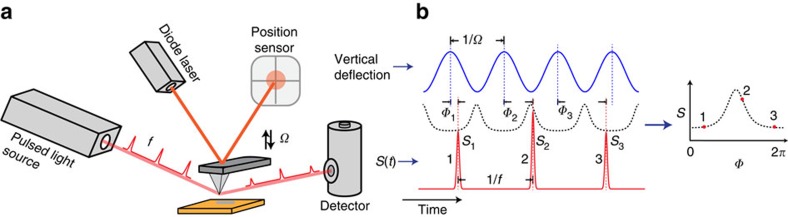
Phase-domain sampling of s-SNOM with the pulsed source. (**a**) An illustration of s-SNOM detection with a low-repetition-rate light source. The pulsed radiation of repetition rate *f* is focused on the tip and sample region of the AFM. The scattered light is converted into pulsed electric signals by an optical detector. The vertical position of the tip is read out with a quadrant photodiode to provide the vertical position of the tip at a given instant of the tip oscillation. The Michelson interferometer that is commonly used in s-SNOM is not shown here for the sake of clarity. (**b**) The amplitude of the pulsed signal (red curve) together with the position of the tip (blue curve) are simultaneously logged and processed. The tip oscillation phases *Φ*_1,2,3…_ at individual pulses are calculated. The tip–sample polarizability (dashed curve) that represents the near-field interactions modifies the scattering signal. The pairs of detector signal *S*_*i*_ and tip oscillation phase *Φ*_*i*_ are used to reconstruct *S*(*Φ*).

The detection mechanism involves simultaneous measurements of the intensities of the scattering signals for individual pulses, and correlates them with the mechanical oscillation phases of the tip at the moment the pulses hit the tip. Figure 1 shows the conceptual scheme of the method. The metal-coated AFM tip is driven at its mechanical resonant frequency of *Ω* Hz and harmonically oscillates above the sample. Periodical variations of the distance between the tip and the sample lead to an anharmonic modulation of the effective polarizability of the tip^27^,^36^,^37^. Depending on the moment when the pulse hits the tip, the light is scattered according to the effective polarizability, which is dependent on the tip–sample distance, or equivalently, the instantaneous oscillation phase of the tip. Consequently, the pulsed scattering signal *S* has a dependence on the instantaneous oscillation phase *Φ* of the tip. Then the scattering signal *S* is correlated with the phase *Φ* for a series of pulses from the light source. Once sufficiently large numbers of the pulses are measured, a correlative relationship *S*(*Φ*) between *S* and *Φ* is obtained, which represents the dependence of the strength of the scattering signal from the tip–sample interaction on the phase of the mechanical oscillation of the tip. This approach is conceptually similar to a procedure of sorting spectra with tip–sample distances that was used in the comb-FTIR s-SNOM (ref. 38).

Note that for *S*(*Φ*) to preserve the details of the near-field interaction *Φ* should densely span 0 and 2*π* with small intervals. This condition is achieved if, after a sufficient number of pulses are recorded, the repetition rate *f* of the light source and the tip oscillation frequency *Ω* lack a common divisor, or their greatest common divisor is small. To illustrate this requirement, let us consider a train of laser pulses from the light source of a repetition rate of *f* Hz. The difference of the instantaneous phase Δ*Φ* between two consecutive pulses is spanned as 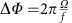
·mod 2*π* , with mod being the modulo operation, which provides the remainder of 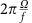 when divided by 2*π*. For a train consisting of *n* pulses, the domain of the set of the phase is 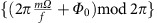, where *Φ*_0_ is the instantaneous tip oscillation phase at the first pulse, and *m* spans from 0 to *n*−1. If a common divisor *q* exists for *f* and *Ω*, the phase domain then consists of discrete values of 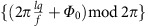, where *l* is from 0 to *f*/*q*. The larger the common divisor *q* is, the fewer phase values are in the phase set. For example, if the tip oscillation frequency is 248 kHz and the laser repetition rate is 10 kHz, the largest common divisor is 2 kHz. If the initial phase *Φ*_0_ is assumed to be 0, the phase domain consists of {0, 0.4*π*, 0.8*π*, 1.2*π*, 1.6*π*}. If the tip oscillation is at 249 kHz, then the phase domain consists of {0, 0.2*π*, 0.4*π*, 0.6*π*, 0.8*π*, *π*, 1.2*π*, 1.4*π*, 1.6*π*, 1.8*π*}, which is twice as dense. In practice, unless specifically engineered, the possibility of the tip oscillation frequency *Ω* and laser repetition rate *f* to have a common divisor, or to have a large common divisor is quite low. If a large common divisor exists, one can avoid it by driving the tip at a small detune within its mechanical resonance, to achieve a densely populated phase domain for *Φ* between 0 and 2*π*.

The next step is to quantitatively extract near-field responses from *S*(*Φ*). The curvature of *S*(*Φ*) can be analysed to obtain anharmonic components which represents the near-field signal. For example, we can fit *S*(*Φ*) within the *Φ* domain of 0 to 2*π* with the form of 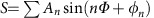 to obtain the coefficient *A*_*n*_ and *ϕ*_*n*_, for *n*≥2. The amplitude coefficient *A*_*n*_ is equivalent to values obtained from lock-in demodulation. Alternatively, we can construct an infinite sequence of *S*(*Φ*) for *Φ* between −∞ and ∞ by repeating *S*(*Φ*) and perform Fourier analysis to obtain the non-fundamental harmonic components. A series of harmonics at the multiple of 1/2*π* frequencies with both amplitude *A*_*n*_ and phase *ϕ*_*n*_ can be extracted from *S*(*Φ*). One of the non-fundamental harmonic components is used to represent the near-field response, analogous to that of lock-in detection.

### Protocol of the phase-domain sampling

**Figure 2:**
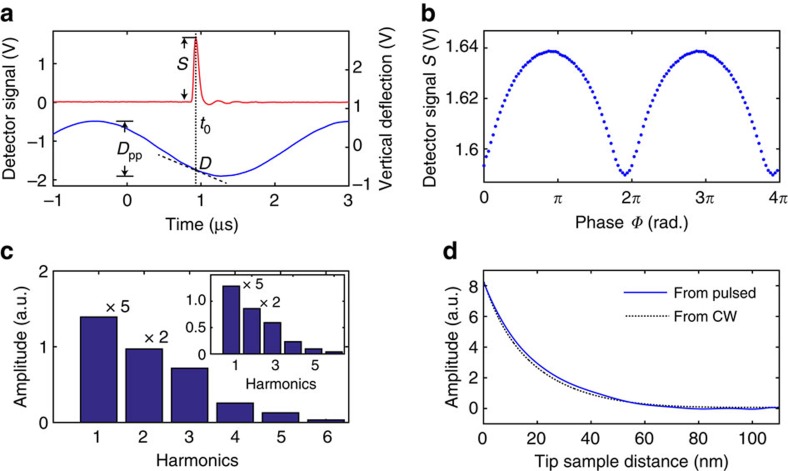
Experimental validation of s-SNOM with the pulsed source. (**a**) The electric signal of a pulse from the detector (red curve) with simultaneously co-registered time-varying vertical deflection of the tip (blue curve). (**b**) Reconstructed *S*(*Φ*) by correlating the amplitude *S* and the calcualted tip oscillation phase *Φ* from 2 × 10^5^ pulses recorded similar to **a**. For better illustration, the phase domain for *Φ* between 0 and 4*π* is shown, which is created by repeating *Φ* in two periods. (**c**) Amplitudes of the harmonics from Fourier analysis of a reconstructed waveform of *S*(*Φ*). The inset is the direct measurement with lock-in demodulation of continuous-wave laser under otherwise the same condition. The first and the second-harmonic amplitudes are scaled down by 5 and 2, respectively. The unit a.u. of vertical axis stands for arbituary unit, same for **d**. (**d**) A comparison of near-field interaction curves reconstructed from the pulsed light source (blue curve) and from the continuous wave light source (dashed curve) with lock-in detection.

In our proof-of-principle experiment, we use a quantum cascade laser (QCL) operated in the pulsed mode with 20 ns duration and test the feasibility of the method with a 30 nm radius Pt-coated tip on a gold substrate. Figure 2a shows the tip–sample scattering signal from the infrared detector (red) of a pulse, and co-registered tip vertical deflection signal (blue). The measured electric signal has a full-width at half-maximum of 125 ns, which is a convolution between the pulse duration and the detector response function. The peak intensity of the signal from the detector is used as the signal *S* (we have also tested using the peak area as the *S*, which yields similar results). In our AFM, the position of the tip is detected by the deflection of light-reflecting cantilever with a quadrant photodiode. The vertical deflection signal *D*(*t*_*i*_) from the quadrant photodiode is simultaneously registered with the detector signal *S*(*t*_*i*_) at the time *t*_*i*_, when the pulse hits the tip and sample. The peak-to-peak vertical deflection *D*_pp_ of the tip oscillation is also recorded, or directly obtained from the AFM controller. The slope of the tip oscillation 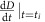 at the measurement time is calculated through sampling of the vertical deflection signal with time. The knowledge of the slope 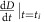 is necessary to remove the duality of the two possible values of the mechanical phase for a given value of tip vertical deflection. The mechanical oscillation phase of the tip is calculated with equations (1) and (2).


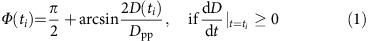



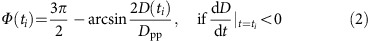


The sign of the slope 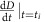 is used to determine whether the phase *Φ*(*t*_*i*_) is between the branch of 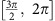 and 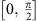, or the branch of 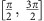, as the range of arcsin function covers only [−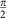, 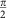] rather than a full range of [0, 2*π*]. Then the pair of *S*(*t*_*i*_) and *Φ*(*t*_*i*_) are correlated to obtain the relation of *S*(*Φ*) for the measurement of the pulse. A sufficiently large number of pulses are measured and averaged in the phase-domain for the near-field reconstruction.

### Proof-of-concept

Figure 2b shows the obtained phase-signal *S*(*Φ*) relationship binned in 64 phase values between 0 and 2*π*, averaged from 2 × 10^5^ pulses with 5 nJ per-pulse energy from the QCL in pulsed mode of 10 kHz repetition rate. The nonlinear distance dependence of the tip–sample near-field interaction causes the curvature to deviate from the sinusoidal shape. Supplementary Fig. 1 shows the reconstruction with 2 × 10^4^ pulses as comparison for the dependence of signal on the number of pulses, and it shows that 2 × 10^4^ pulses are sufficient for reconstructing *S*(*Φ*) from strong near-field interactions on gold substrate. Fourier analysis on *S*(*Φ*) with the procedure described above is shown in Fig. 2c. A series of non-fundamental harmonic components are obtained, which are a characteristic of the near-field interaction. Lock-in demodulations of direct continuous-wave measurement are shown as the inset for comparison. In addition, the amplitude and phase of a series of harmonics from the Fourier analysis of *S*(*Φ*) can also be utilized to obtain the distance dependence of the tip–sample near-field interaction with a reconstruction method recently described in literature^39^. The 1/e decay range of the near-field interaction is found to be 19.3 nm, which is consistent with the reconstruction interaction curve of the same sample using continuous-wave lasers (dashed curve). Continuous-wave measurement is obtained with 164 ms lock-in time constant with 80 mW laser power from the QCL in continuous-wave mode. It corresponds to a total 13.1 mJ in the acquisition of the signal. In comparison, the total photon energy of 2 × 10^5^ number of 20 ns pulses of is ∼1 mJ, which is one order of magnitude smaller than that from the continuous wave. The reduction of the total energy leads to an increase in noise, manifested as the slight wavy feature when the separation between the tip and the sample is large, as shown in Fig. 2d.

### Measurements on boron nitride nanotubes and graphene

**Figure 3:**
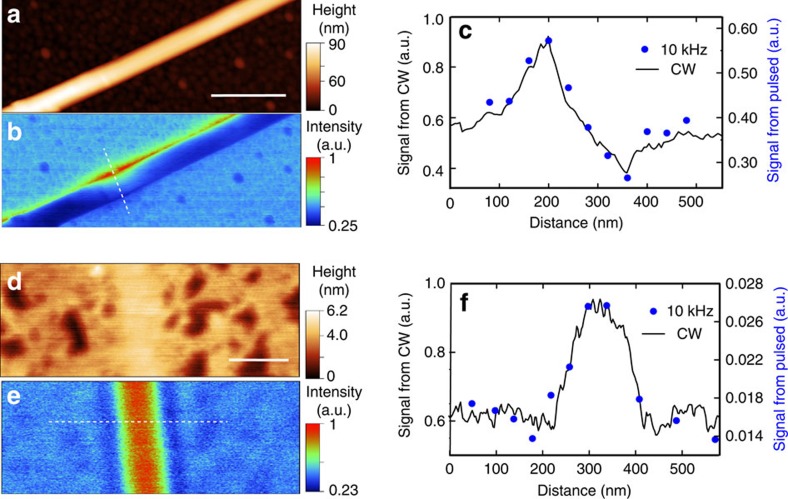
Spatial responses with low-repetition-rate source. (**a**) AFM topography of a BNNT of 70 nm diameter. The scale bar is 500 nm, same for **b**. (**b**) The s-SNOM image at the second-harmonic demodulation obtained with QCL operating in the continuous mode at 1,405 cm^−1^. a.u. stands for the arbitrary units, same for **c**,**e**,**f**. (**c**) The comparison between the s-SNOM responses of the BNNT with QCL in continous-wave (CW) mode (black continuous curve) vs. near-field signal profile from the phase-domain sampling with the QCL operated in 10 kHz pulsed mode (blue dots). The location of the profile is shown as the white dashed line in **b**. 2 × 10^5^ pulses are acquired to extract the near-field response per point. (**d**) AFM topography of graphene heterostructure formed by a crease of partially folded monolayer graphene. The scale bar is 200 nm, same for **e**. (**e**) The s-SNOM image at the second-harmonic lock-in demodulation obtained by QCL in CW mode at 1,590 cm^−1^. (**f**) The comparison between the s-SNOM responses of the graphene crease with CW source (black continuous curve) and near-field signal profile from the pulsed mode at 10 kHz repetition rate mode (blue dots). The location of the profile is shown as the white dashed line in **e**. 2 × 10^5^ pulses are acquired to extract the near-field response per point.

The low-repetition-rate detection mechanism preserves the spatial resolution of the s-SNOM, as the spatial dependence of the scattering signal is independent of the temporal duration of the pulsed light source. We have carried out the near-field experiments on polariton supporting materials of a boron nitride nanotube (BNNT) and a graphene crease to spatially test the correspondence between the s-SNOM image and near-field signal obtained from the method of low-repetition-rate pulses. Figure 3a,b shows the AFM topography of a BNNT on a gold substrate and s-SNOM image at the frequency of 1,405 cm^−1^ with second-harmonic demodulation. The BNNT can support surface phonon polaritons that lead to spatial variations of the s-SNOM signal in the Reststrahlen band of BN in the mid-infrared^40^,^41^. The profile of extracted near-field response from the second-harmonic component of *S*(*Φ*) with the low-repetition-rate method (at 10 kHz) across the BNNT is shown as blue dots in Fig. 3c. The response is obtained along the white dashed line in Fig. 3b. The profile from the s-SNOM image in Fig. 3b obtained with continuous-wave mode of the QCL is shown as the reference (black curve). The two methods show good agreement. Figure 3d,e show the AFM topography and s-SNOM image of a crease formed by a folded monolayer graphene on a SiO_2_ substrate. The crease made of three folded graphene layers shows stronger s-SNOM response than the monolayer graphene at the infrared frequency of 1,590 cm^−1^. The extracted near-field response of second-harmonic component of *S*(*Φ*) from the pulsed method with 10 kHz repetition rate and 20 ns pulse duration is shown in Fig. 3f as blue dots. The profile is obtained along the white dashed line in Fig. 3e. The s-SNOM response of second-harmonic demodulation from the continuous-wave mode of QCL is included for comparison (black curve), which agrees with the response obtained from the phase-domain sampling. The discrepancy between two profiles is likely due to the drifting of the tip position on the sample during the measurement.

### The phase-domain sampling with random repetition-rate pulses

**Figure 4:**
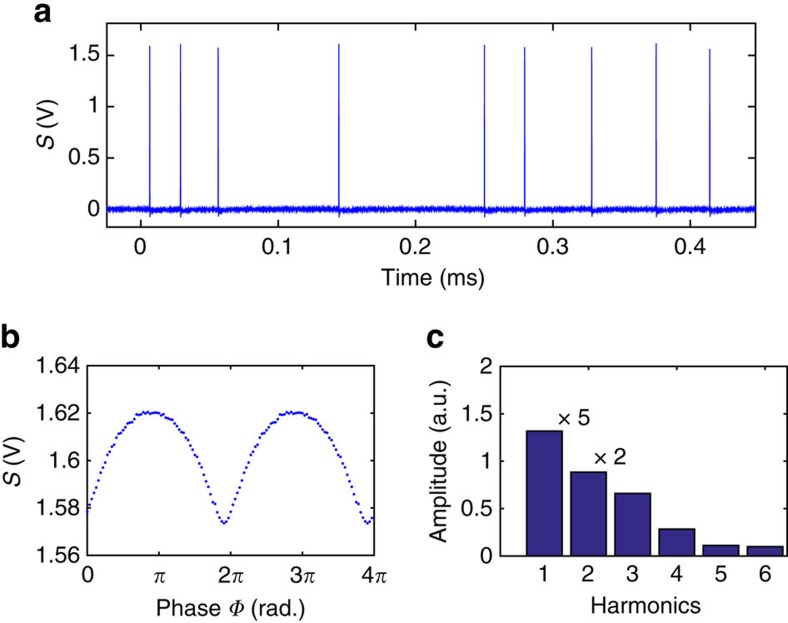
Near-field signal with irregular repetition-rate pulses. (**a**) Detector responses for a segment of a pulse sequence with irregular timing. (**b**) Reconstruction of *S*(*Φ*) from experimental measurement of 2 × 10^4^ pulses from the irregular timing sequence shown in **a**. The signal is obtained with Pt-coated tip on a rough gold substrate, taken at 1,590 cm^−1^ infrared frequency. (**c**) Corresponding harmonic components from the reconstructed waveform *S*(*Φ*) in **b**. It can be seen that the random timing of the pulses does not affect the performance of the phase-domain sampling. a.u. stands for arbitrary unit. The first and second-harmonic amplitudes are scaled down by 5 and 2, respectively.

The principle of the detection method allows its adaptation to various repetition rates of the pulsed light source, from a low-repetition rate well below the tip oscillation frequency to moderately high frequency below the critical sampling rate of the lock-in detection determined by the Nyquist–Shannon theorem. We have tested the method with repetition rates of 1, 10, 100 and 400 kHz repetition rate. (Supplementary Fig. 2) They provide very similar shapes on the s-SNOM waveform and harmonic extractions. In principle, the upper limit of the workable repetition rate of the method is determined by the response function of the optical detector, rather than the actual oscillation frequency of the tip. Moreover, as long as the data acquisition from the detector and the position sensor of the s-SNOM tip is simultaneously registered, detection mechanism does not require the laser to have a well-defined repetition rate. Figure 4 shows the experimental result that demonstrates this attribute. The QCL is operated with an external trigger from a sequence of noise waveform generated by a function generator. The pulses from the QCL in this mode have random timing (Fig. 4a) and lack a well-defined repetition rate. The phase-domain sampling method relies on the instantaneous mechanical phase of the tip, so random timing between pulses does not affect the readout of the mechanical phase of the tip for individual pulses. Therefore, the reconstruction of the *S*(*Φ*) (Fig. 4b) from the measurement of a sequence of pulses and subsequent harmonic extraction (Fig. 4c) are not affected. Had the lock-in detection been used, the noise that was associated with random timing of the pulses would enter into the Fourier analysis and affect the extraction of the near-field signal, in addition to the limitations posed by the Nyquist–Shannon theorem. Pulsed light sources prone to high jitter are still compatible with this method, such as from a regenerative amplifier where the operation of pulse-picking Pockels cell is not perfectly synchronized with the timing of the seeding oscillator.

## Discussion

In traditional s-SNOM techniques with lock-in detection, the detector signal is sampled in the time domain with equal intervals, which is subject to the restriction of the Nyquist–Shannon theorem. Supplementary Figs 3 and 4 show s-SNOM images with a laser source with different repetition rates. The near-field contrast vanishes for the repetition rate lower than the tip oscillation frequency. In our method, the effective signal sampling is done in the phase domain, given the periodical nature of the near-field interaction due to the harmonic oscillations of the tip. In a way, the method bypasses the restriction of the Nyquist–Shannon theorem, as it is no longer continuous sampling in the time domain. On the other hand, the density of the phase domain in our method is still subject to the Nyquist–Shannon theorem, as the phase domain should be dense enough to reflect the curvature of anharmonicity in *S*(*Φ*) with Fourier analysis. The sampling of individual pulses is also subject to the Nyquist–Shannon theorem. The data acquisition device should be fast enough to faithfully capture the height of the signal peaks without aliasing, or properly triggered with the timing of the pulsed light source^25^. On the other hand, the conventional lock-in detection can be considered as a special case of phase-domain sampling, the lock-in amplifier continuously samples the signal to sequentially populate the phase domain for harmonic demodulation. In comparison, the requirement of sequential sampling of the phase domain is relaxed with the proposed method, therefore it even allows utilization of irregular repetition-rate pulses.

The proposed method could potentially enable s-SNOM with kilohertz ultrafast femtosecond amplifier system with high-peak power in research labs. The table-top 10 kHz femtosecond amplifier system of 1 W output at 100 fs duration has a per-pulse power of 1 × 10^9^ W. In comparison, the table-top 80 MHz femtosecond oscillator system of the same average power and pulse durations has a per-pulse power of 1.25 × 10^5^ W, which is about four orders of magnitude less than that of the amplifier system. The high-peak power from low-repetition-rate ultrashort pulses is suitable to drive nonlinear optical processes, to study strong field light–matter interactions and perform time-resolved pump-probe experiments. The method enables simple interfacing between the existing kilohertz repetition rate laser sources and s-SNOM to study these phenomena at the nanoscale with 10–20 nm spatial resolution. As far as we know, the femtosecond pump-probe s-SNOM investigations have been carried out with 80 MHz (refs 34, 35), and 1 MHz (ref. 29) repetition rate. The possible utilization of high-power pulsed lasers from low-repetition rate kHz femtosecond amplifier systems will facilitate the light–matter interaction in the pump-probe s-SNOM studies. In addition, for femtosecond pulsed laser with broad bandwidth, the mechanism of the method is compatible with the technique of nano-FTIR (refs 24, 42), which acquires spectra through Fourier transform of interferograms. Supplementary Fig. 5 shows a segment of an interferogram obtained from the low-repetition-rate method with QCL operating at 10 kHz, while the tip oscillates harmonically at ∼250 kHz.

The proposed method can enable s-SNOM operation with tips of high mechanical resonant frequencies with low-repetition-rate pulsed light sources, as opposed to using a tip with low mechanical resonant frequency to reduce the critical sampling rate as required by the Nyquist–Shannon sampling theorem. A cantilever with high mechanical resonant frequency is more rigid than a cantilever with low mechanical resonant frequency, given comparable reduced masses of the cantilevers. The mechanical oscillation from a rigid cantilever is more harmonic than that from a soft cantilever in the tapping mode, as the gradient of the intermolecular forces between the tip and sample has less an effect on the motion of the rigid cantilever than a soft cantilever. This is beneficial for s-SNOM operations, as non-harmonic components from the scattering signal should ideally be from the tip–sample near-field interaction, rather than the anharmonic motion of the tip itself^43^,^44^.

The main limitation of this method is that the speed of the measurement is bound by the number of available pulses in unit time. For a light source with lower repetition rate, this would mean inevitably longer acquisition time. For example, at a repetition rate of 10 kHz, if 2 × 10^4^ pulses are needed to reconstruct the near-field interaction, the acquisition time of per pixel would be 2 s, which is fast enough for a line scan across a feature of interest, but it is very slow for generation of a full frame s-SNOM image (for example, a 128 × 128 pixels image would take 9.1 h). On the other hand, this limitation is alleviated with the increase of the repetition rate. At 100 kHz repetition rate, the scan is ten times faster, assuming the hardware data acquisition and processing speed can keep up with the increased number of pulses. In our current implementation, the signal processing is done at software level after hardware acquisition, which limits the processing speed. The method could be further improved with the utilization of field-programmable gated array device with fast on-board processing^45^, to increase the acquisition and processing speed.

Another limitation of the method is that the pulse-to-pulse stability of the light source affects the quality of the reconstructed *S*(*Φ*). Since the near-field interaction is small compared with the far-field background, the pulse-to-pulse energy fluctuation is likely to be the dominant source of noise. The r.m.s. pulse-to-pulse power fluctuations of our QCL source are 1.4 and 2.7% respectively for two chips (Supplementary Fig. 6). In comparison, the second-harmonic demodulated component of the near-field signal from the gold substrate is about 0.8% of the averaged scattering signal in the measurement of Fig. 2. In the presence of large pulse-to-pulse energy fluctuations, more pulses are inevitably needed for averaging to obtain *S*(*Φ*) for signal extraction, thus increasing the required acquisition time. If the pulse-to-pulse power fluctuation could be reduced to one third of the current level, the required acquisition time would be reduced to one ninth, which would considerably shorten the acquisition time. In fact, several commercially available solid-state regenerative amplifiers have pulse stability better than 0.75%, which should provide better performance than the QCL. In addition, to overcome this limitation, a reference detector setup can be implemented to account for the pulse-to-pulse energy fluctuation of the light source, similar to that of the dual-beam light absorption spectroscopy (Supplementary Fig. 7). If the pulse-to-pulse power fluctuation is accounted, the number of pulses required for the reconstruction of *S*(*Φ*) will decrease, which will also lead to an increase of the measurement speed. For future applications in pump-probe or nonlinear optical s-SNOM with low-repetition-rate light source, one can in principle utilize a sophisticated reference scheme that considers the order of the nonlinear interaction to account for the fluctuations. For example, if a second order nonlinear process is considered, one can implement a far-field reference with a nonlinear crystal (for example, beta barium borate) to account for the fluctuations on the power, durations, or optical phases of the involved pulses. Two experimental schemes are included in Supplementary Fig. 8 and Supplementary Note 1 on possible implementations of nonlinear ultrafast s-SNOM with the phase-domain sample approach with pulse-to-pulse fluctuation compensation using a far-field reference.

Another possible limitation of the method is that it does not work with the popular pseudo-heterodyne technique^46^. Pseudo-heterodyne technique is a double-demodulation lock-in technique in s-SNOM that provides phase information. Although, one could imagine the implementation of pseudo-heterodyne through expansion of phase domain with both the tip oscillation phase and the reference mirror modulation phase, and perform Fourier transform to obtain the side band harmonics to recover the equivalent pseudo-heterodyne signals. On the other hand, the phase-domain sampling method is compatible with the homodyne technique that provides similar capability as the pseudo-heterodyne technique. By utilization of a Michelson interferometer to adjust and maintain the optical phase of the reference optical field at specific values, the reference field can selectively amplify the components of the near-field response. By adjusting the homodyne field to be in-phase or *π*/2 out of the phase, the real or imaginary part of the near-field responses can be retrieved in homodyne detections. In Supplementary Fig. 9, we demonstrate the use of the low-repetition-rate method with quadrature-phase homodyne technique to obtain the real and imaginary parts of the near-field responses.

The phase-domain sampling method enables scattering-type s-SNOM with low-repetition-rate pulsed light sources. It bypasses the requirement of pulse repetition rate imposed by the Nyquist–Shannon sampling theorem for lock-in detections. Experimental results show the reconstruction method for the pulsed source is equivalent to the traditional lock-in detection mechanism with continuous-wave light source. The method expands the availability of light sources for scattering-type s-SNOM and potentially enables the utilization of coherent radiation derived from the pulsed laser from popular amplified ultrafast laser systems for the nanoscale spectroscopic investigations that require high instantaneous optical powers in the field of nonlinear nano-optics and pump-probe s-SNOM.

## Methods

### Experimental setup

The experimental setup is shown in Supplementary Fig. 7, which consists of an AFM (Multimode, Bruker), an infrared light source, a custom-built, stabilized Michelson interferometer and an infrared detector. A QCL (Daylight Photonics) is operated in the either pulsed mode or continuous-wave mode as the infrared light source. In the pulsed mode experiment, repetition rates of 1, 10, 100 and 400 kHz were used. The pulse duration of the QCL was set to 20 ns. A metal-coated tip (Arrow NCPt, Nanoworld) oscillates at its mechanical resonance frequency of about 246.9 kHz. The pulsed laser radiation from the QCL is coupled to a BaF_2_ beam splitter of the interferometer. One portion of the laser radiation is reflected to the tip region by an off-axis parabolic mirror with a numeric aperture of 0.25. The tip scattered light is collected and detected by an infrared detector (KLD, Kolmar Tech). The signal from the detector is measured by a two-channel data acquisition card (NI PXI-5122, 100 M sample per second National Instruments) simultaneously with the tip vertical deflection signal from the quadrant photodiode of the AFM. The amplitude and time derivative of the tip vertical deflection signal are used to calculate the instantaneous oscillation phase of the tip *Φ*. The instantaneous oscillation phase *Φ* is correlated with simultaneously measured scattering signal *S* from the infrared detector to generate *S*(*Φ*).

### Data processing

A MATLAB code is written to average the relationship between *S*(*Φ*) and 64 equally spaced phase points, and perform Fourier analysis on the non-fundamental harmonic to obtain a series of harmonic components and phases.

### Data availability

The data that support the findings of this study are available from the corresponding author upon reasonable request.

## Additional information

**How to cite this article:** Wang, H. *et al*. Scattering-type scanning near-field optical microscopy with low-repetition-rate pulsed light source through phase-domain sampling. *Nat. Commun.* **7,** 13212 doi: 10.1038/ncomms13212 (2016).

## Supplementary Material

Supplementary Information Supplementary Figures 1-9, Supplementary Note 1 and Supplementary Reference
