# Supplementary material for: Scattering-type scanning near-field optical microscopy with low-repetition-rate pulsed light source through phase-domain sampling
Source: Nat Commun. 2016 Oct 17;7:13212. doi: 10.1038/ncomms13212 (PMC5071641; doi:10.1038/ncomms13212)
Supplement: Supplementary Information — Supplementary Figures 1-9, Supplementary Note 1 and Supplementary Reference [file ncomms13212-s1.pdf]

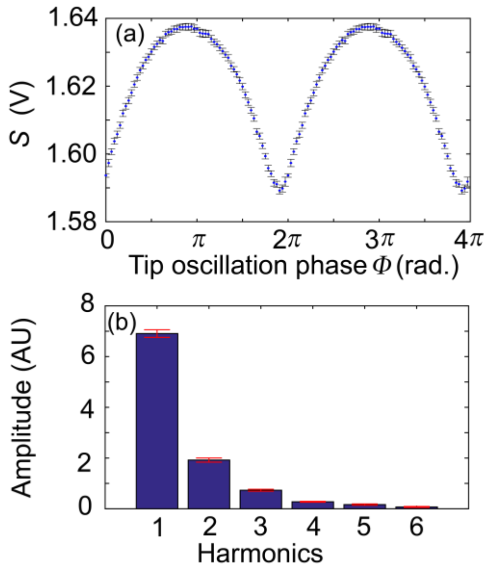

Supplementary Figure 1. Near-field responses from the phase-domain sampling of 2×10^4 pulses

(a) s-SNOM waveform $S(\Phi)$ obtained from the phase-domain sampling measurement of 2×10^4 pulses under the same conditions as Fig. 2 in the main text. The error bars at each point is 95% confidence limit derived from the detector signals of many pulses that fall in one same phase interval. 64 phase intervals spanning equally from 0 to 2π are used. (b) The averaged harmonic values from Fourier analysis of waveforms of ten sets of 2×10^4 pulses. The error bars are the 95% confidence limit derived from ten datasets, and each dataset is obtained through phase-domain sampling of 2×10^4 pulses. AU stands for arbitrary unit.

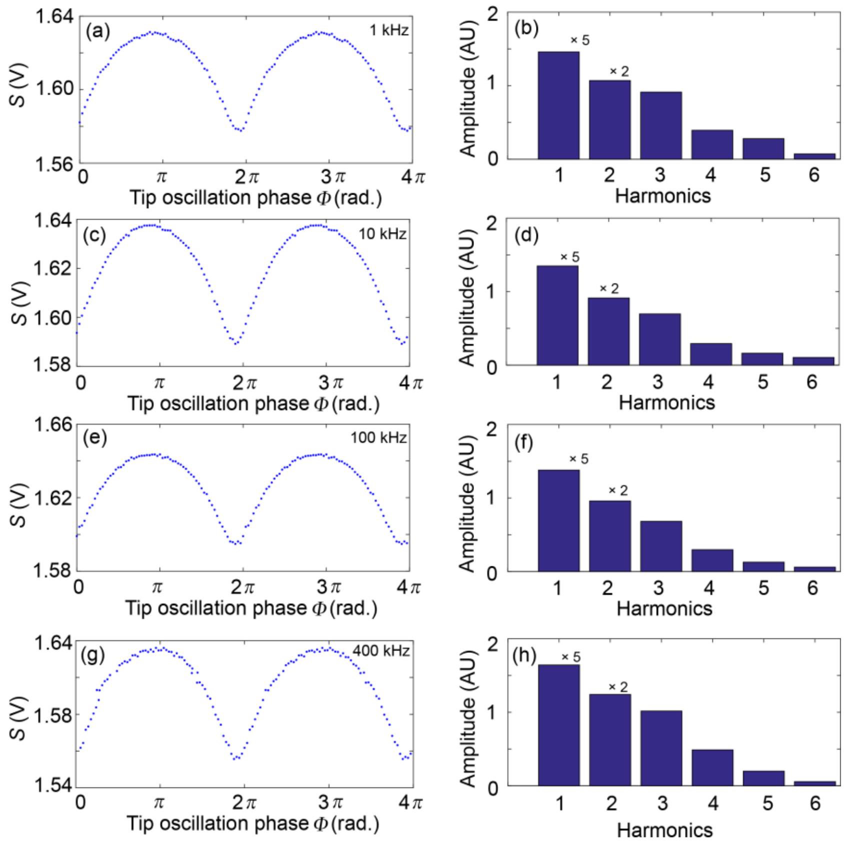

Supplementary Figure 2. Reconstructed $S(\Phi)$ at different laser repetition rates

(a) s-SNOM waveform $S(\Phi)$ at 1 kHz. (b) Fourier components of (a). AU stands for arbitrary unit, same for (d), (f) and (h). The first and second harmonic amplitudes are scaled down by 5 and 2, respectively. The same operation is done for (d), (f) and (h). (c) s-SNOM waveform $S(\Phi)$ at 10 kHz. (d) Fourier components of (c). (e) s-SNOM waveform $S(\Phi)$ at 100 kHz. (f) Fourier components of (e). (g) s-SNOM waveform $S(\Phi)$ at 400 kHz. (h) Fourier components of (g). They all show anharmonic shape, characteristics of near-field interaction between the sample and the s-SNOM tip. The relation $S(\Phi)$ from 1 kHz, 10 kHz and 100 kHz show the same shapes and levels of noise. The relation $S(\Phi)$ from 400 kHz shows additional noise, possibly due to the limitation of the stability of the quantum cascade laser operated at a high repetition rate.

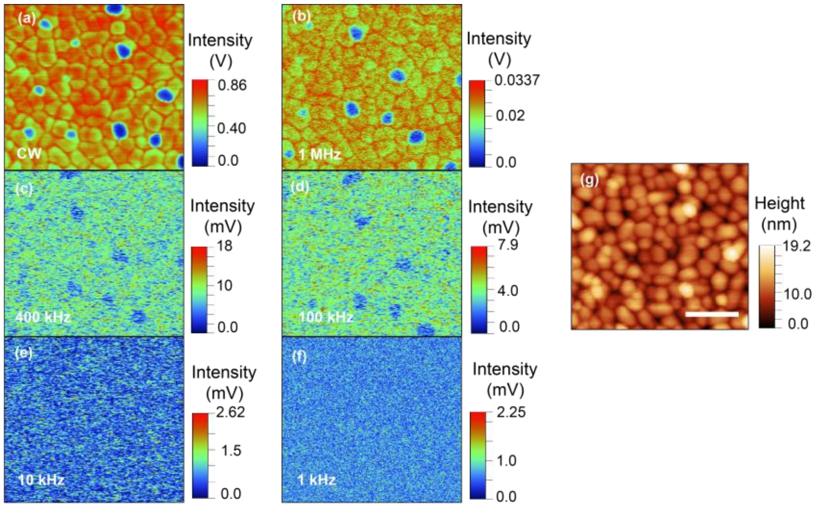

Supplementary Figure 3. s-SNOM images of a rough gold substrate obtained at different laser repetition rates

(a) The s-SNOM image of a rough gold substrate with small dirt particles, obtained with continuous wave (CW) infrared light source from the quantum cascade laser. Second harmonic demodulation is used. Lock-in detection was done by a lock-in amplifier (HF2Li Zurich Instruments). (b) s-SNOM image with the quantum cascade laser operated at 1 MHz repetition rate of 10% duty cycle. The quality of the s-SNOM image is drastically reduced, as it is close to the critical sampling rate set by the Nyquist-Shannon sampling theorem. The maximal signal (0.0337 V) is reduced from that of CW mode (0.86 V) by a factor more than the reduction of the laser power (10%). (c) The s-SNOM image with the quantum cascade laser operated at 400 kHz repetition rate of 4% duty cycle. The image shows poor contrast and the signal reduction is much more than the reduction of the laser power, because the second harmonic signal is under-sampled at 400 kHz, for a tip of ~ 250 kHz mechanical oscillation frequency. (d) the s-SNOM image with laser operated at 100 kHz repetition rate of 1% duty cycle. Further reduction of the signal is observed. (e-f) s-SNOM with the pulsed laser operated at 10 kHz and 1 kHz repetition rate is unable to provide any contrast, as such low repetition rate is unable to generate lock-in detectable signals at the harmonic of the tip mechanical oscillation frequency. The topography of the rough gold substrate is shown in (g). The scale bar is 200 nm for all. The scan size is 640 × 640 nm^2. The lock-in time constant is 3 ms for the measurement in (a-f).

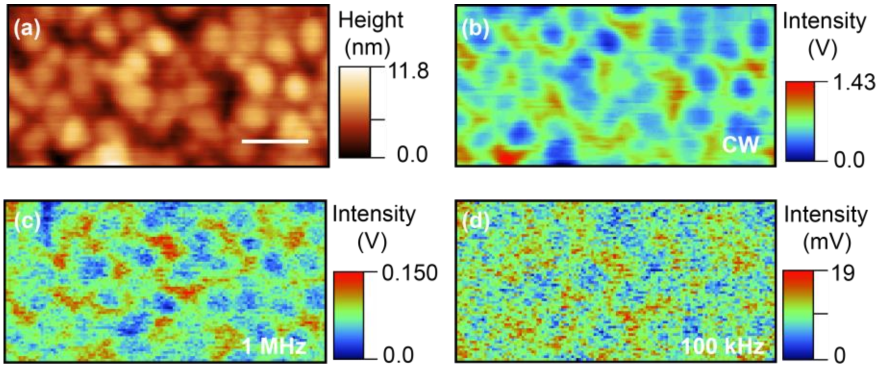

Supplementary Figure 4. Comparison of s-SNOM image quality between continuous wave and pulsed laser in lock-in detection with the same total photon energy

(a) Topography image of an area of a rough gold substrate. The scale bar is 100 nm, same for (b) to (d). (b) s-SNOM image of the area obtained with continuous wave QCL with lock-in time constant set at 0.4 ms. Infrared frequency was set at 1585 cm^{-1}. Second harmonic demodulation was used. The tip oscillation frequency is ~280 kHz. (c) s-SNOM image obtained with the QCL operating in the pulsed mode with 1 MHz repetition rate and 20 ns pulse duration. The lock-in time constant is chosen to be ~20 ms. Significant reduction of the image quality is observed. (d) s-SNOM image obtained with QCL operating at 100 kHz repetition rate and 20 ns pulse duration. The lock-in time constant was chosen to be ~200 ms. It can be seen that the quality of the image is reduced further. The lock-in time constants, 0.4 ms, 20 ms, and 200 ms were chosen to have the same total photon energy per pixel in the acquisition of the s-SNOM images for the continuous wave and pulsed mode. This measurement demonstrates that even with the same total photon energy per pixel, the distribution of the photon energy in time is critical for lock-in detection. A continuous wave light source is better for lock-in detection, whereas pulsed light source with low repetition rate is difficult for lock-in measurements in s-SNOM below the Nyquist-Shannon frequency.

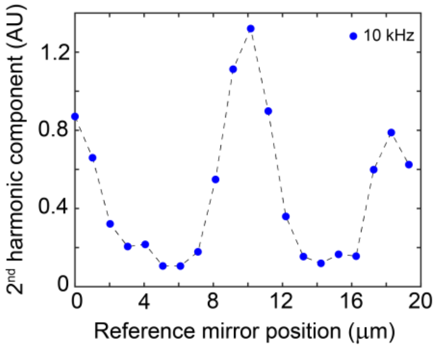

Supplementary Figure 5. A segment of an interferogram obtained by phase-domain sampling

The interferogram of second harmonic components obtained through the phase-domain sampling method, blue dots are obtained with quantum cascade laser operated at 10 kHz pulsed mode centered at 1370 cm^{-1} infrared frequency, then they are connected by the dashed line to show the trend. This interferogram is obtained by translating the position of the reference mirror in the Michelson interferometer. AU stands for the arbitrary unit.

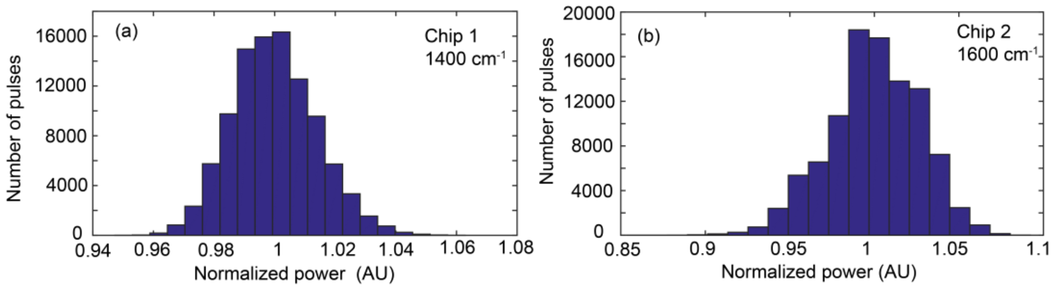

Supplementary Figure 6. Estimation of the power stability of the quantum cascade laser in this study

Totally 1×10^5 pulses are measured with an IR detector for each chip. (a) The r.m.s. noise of the QCL chip of 1400 cm^{-1} is 1.4%. AU stands for the arbitrary unit, same for (b). (b) The r.m.s. noise of the QCL chip of 1600 cm^{-1} is 2.7 %. The noise amplitude is larger than the signal generated by the near-field interactions, which is estimated to be less than 0.8% in the second harmonic demodulation for the measurement in Fig. 2 of the main manuscript. This explains why a large number of pulses are needed for acquisition.

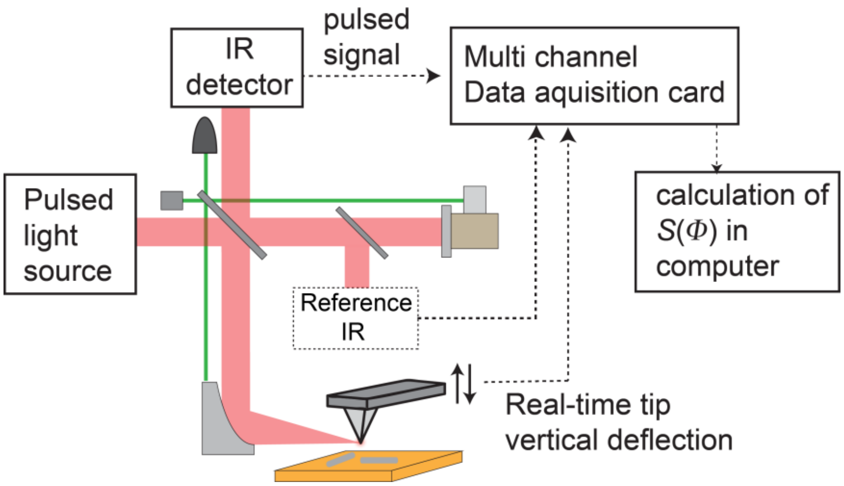

Supplementary Figure 7. The experimental setup of the custom-built s-SNOM apparatus

It consists of a pulsed light source, a stabilized Michelson interferometer, and an atomic force microscope built according to literature[1]. The interferometer is used to adjust the homodyne phase of the measurement. The pulsed signal from the infrared detector is simultaneously recorded with the real-time tip vertical deflection signal with a two-channel data acquisition card. At the moment, our data acquisition card (NI-PXI 5122) only allows simultaneous collection of two channels. A further improvement would be to use a three-channel data acquisition device that can include an additional channel for a reference infrared detector to account for the pulse fluctuation, thus reducing the noise in $S(\Phi)$.

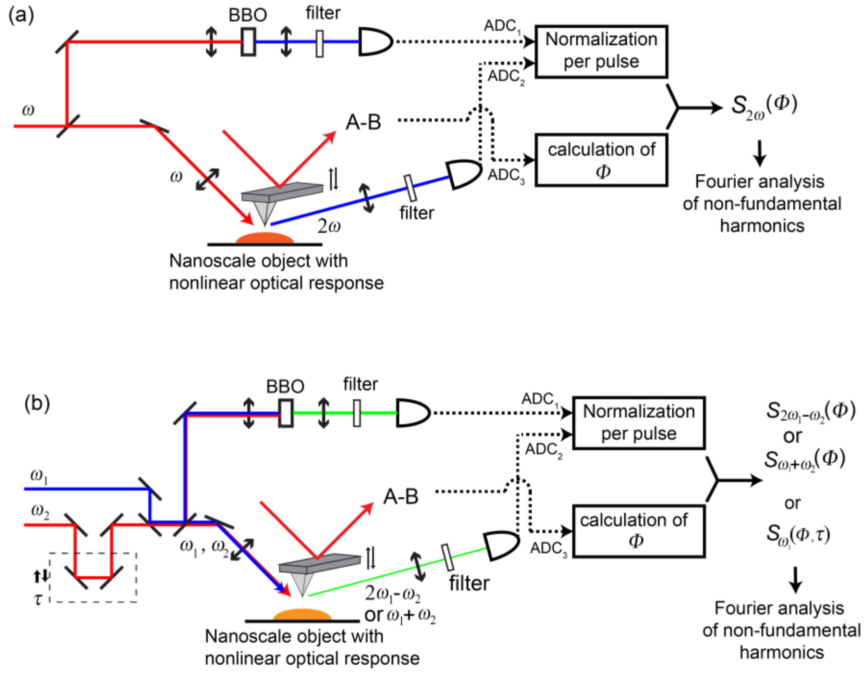

Supplementary Figure 8. Two schemes of implementation for nonlinear s-SNOM with low repetition rate femtosecond laser pulses

(a) Single-colour (central frequency ω) pulsed s-SNOM setup to study second harmonic generation (2ω) from nanoscale objects. (b) Two-colour (central frequencies ω_1 and ω_2 pulsed s-SNOM setup to study nonlinear optical responses from nanoscale objects. The responses can be four-wave-mixing ($2\omega_1 - \omega_2$), sum frequency generation ($\omega_1 + \omega_2$), or transient absorption/transient grating type interactions ($\omega_1 + \omega_2 - \omega_2$). In these experiments, a separate, far-field reference medium that supports nonlinear optical interaction is used, e.g., a beta barium borate crystal (BBO), to generate nonlinear optical signal of the same type as the interaction of interest from the nanoscale objects. The signal from the nonlinear crystal is used as the reference to be simultaneously registered by a multi-channel data acquisition card with the scattering signal from the tip-sample region through two simultaneous sampling analog to digital converters (ADCs). Proper bandpass optical filters are used to allow only the optical frequencies of interest (e.g. 2ω for second harmonic generation, or $2\omega_1 - \omega_2$ for four-wave-mixing) to reach the two simultaneously sampled detectors. The ratio between simultaneously acquired signals from the tip/sample region and the reference BBO crystal are taken as the normalized signal S. The power fluctuation or profile changes from pulse to pulse are cancelled out in the normalized signal from the two simultaneously acquired signals. The normalized scattering signal S is co-registered with the mechanical phase Φ of the cantilever to form the $S(\Phi)$ waveform at the nonlinear optical frequency, and similar treatment describe in the main manuscript is used to

recover the near-field contribution from the normalized signal $S(\Phi)$ Subsequent Fourier analysis on the non-fundamental harmonic curvatures of $S(\Phi)$ should provide the near-field components of the nonlinear optical interaction. This scheme allows extraction of responses of nonlinear optical interactions that are generated due to the presence of the s-SNOM probe at the proximity of the nanoscale object. The nonlinear optical response directly from the sample surface or directly from the cantilever surface should not contribute to the non-fundamental harmonic in $S(\Phi)$, similar to the signal extraction mechanism in the current (linear) s-SNOM measurements. The non-fundamental harmonic components in $S(\Phi)$ correspond to the interactions that are enhanced due to the proximity or field enhancement of the metallic tip, therefore they possess high spatial frequencies and lead to the high spatial resolution in s-SNOM technique. For more details, see Supplementary Note 1.

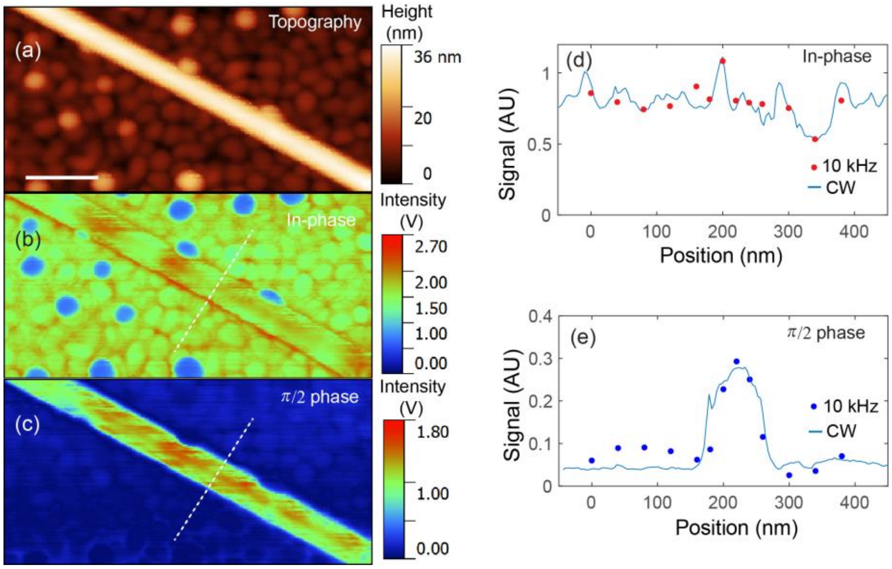

Supplementary Figure 9. Near-field responses under quadrature phase homodyne conditions by the phase-domain sampling method with low repetition rate lasers

(a) The topography of a boron nitride nanotube. The scale bar is 200 nm, same for (b) and (c). (b) s-SNOM image of the boron nitride nanotube obtained with the continuous wave infrared laser with frequency at 1395 cm^{-1} under in-phase homodyne condition. Second harmonic demodulation is used. 3 ms lock-in time constant is used. The s-SNOM signal with in-phase homodyne corresponds to the real part of the near-field response. (c) s-SNOM image of the nanotube obtained with the same laser under quadrature phase ($\pi/2$ phase) homodyne condition. The s-SNOM signal with $\pi/2$ phase homodyne corresponds to the imaginary part of the near-field response. The in-phase and $\pi/2$ phase homodyne conditions were determined with a procedure described in literature.[1] (d) Reconstructed near-field response under in phase homodyne condition (red dots) along the white dashed line shown in (b). The profile from the continuous wave near-field measurement is shown as reference (blue curve). AU stands for arbitrary unit, same for (e). (e) Reconstructed near-field response under $\pi/2$ phase homodyne condition (blue dots) as the same line as (d). The profile from the continuous wave near-field measurement is shown as reference (blue curve). 2×10^5 pulses are acquired for each points for the phase-domain sampling. The measurement shows that the time-domain sampling method with low repetition rate laser is compatible with homodyne techniques.

Supplementary Note 1

The proposed schemes in Supplementary Fig. 8 allow efficient removal of the power and profile fluctuations of the source from pulse to pulse. Take the second harmonic generations as an example. The second harmonic generation in general can be described as $E_{2\omega}(t) = {\chi^{(2)}}_{2\omega:\omega+\omega}\, E_{\omega}(t)E_{\omega}(t)$ in the time domain. ${\chi^{(2)}}_{2\omega:\omega+\omega}$ is the second order susceptibility. $E_{\omega}(t)$ is the electric field of the incident light. The fluctuation or changes of amplitude and temporal profile of the field correspond to the changes in $E_{\omega}(t)$, which will lead to changes of the nonlinear signal at the 2ω frequency. On the other hand, the nonlinear susceptibility ${\chi^{(2)}}_{2\omega:\omega+\omega}$ remains independent of the profile of the fields, as it is phenomenologically a scaling factor that connects the incident field $E_{\omega}(t)$ with the second harmonic field $E_{2\omega}(t)$ without explicit dependence by its definition. ${\chi^{(2)}}_{2\omega:\omega+\omega}$ is a property of the media on which the optical fields are applied. In the far-field nonlinear optical experiment, the samples have specific values of ${\chi^{(2)}}_{2\omega:\omega+\omega}$ that determine the generation efficiency of the second harmonic signal at the input frequency of ω. Note that the $E_{\omega}(t)$ is the propagating electric field in the conventional definition of susceptibility. In the near-field nonlinear optical experiment, the tip and the sample can be collectively defined to have an effective ${\chi^{(2)}}_{2\omega:\omega+\omega}$ that describes the nonlinear optical signal generation efficiency of the collective tip-sample region. The effective nonlinear susceptibility multiplied with the far-field incident fields is defined to be the same as the conventionally defined nonlinear susceptibility multiplied with the strength of the local optical near field The tip-distance dependence of the local electric near-field enhancement is absorbed into the effective ${\chi^{(2)}}_{2\omega:\omega+\omega}$. Such a definition allows the treatment to work with the far-field measurable electric field, rather than the local electric field that has strong spatial variations.

In the proposed scheme, the reference nonlinear crystal, e.g., BBO is used to generate a reference optical signal at the second harmonic frequency 2ω, and measured simultaneously with the signal from the tip and sample for each pulse with a photodetector with band pass filter of frequency 2ω. The profile fluctuations in the incident pulse affect the generation of the reference signal in the BBO crystal in the same way as the generation of the second-harmonic signals from the collective tip/sample region. Therefore, a subsequent normalization (through division) of the detected scattering signal from the tip-sample region and the reference BBO crystal should yield the squared value of the ratio between ${\chi^{(2)}_{\text{scattering}}}_{2\omega:\omega+\omega}$ the effective second-order nonlinear susceptibility of the tip and sample, and ${\chi^{(2)}_{\text{ref}}}_{2\omega:\omega+\omega}$ of the BBO scaled with a coefficient of detection efficiency. In the treatment of the taking the ratio between the tip/sample signal and the reference for individual pulses, the contribution from the incident field is cancelled, and consequently the temporal fluctuations of the source are also cancelled.

In similar cases of the four-wave-mixing or sum frequency generations, we have put a scheme in Supplementary Fig. 9b. It utilizes a reference nonlinear crystal to generate the frequency of the interest for individual pulses. For example, the four-wave-mixing is $2\omega_1 - \omega_2$ and the sum frequency is $\omega_1 + \omega_2$. The generated signal is simultaneously logged with the scattering light from the tip and sample. Similar to the treatment described above, the signal from the tip/sample is divided by the signal from the reference nonlinear crystal to obtain the ratio between the nonlinear susceptibilities of the tip/sample region and the reference crystal. In this case, the temporal fluctuations from the sources are also cancelled, only the ratio of $|\chi^{(3)}_{\text{scattering}}/\chi^{(3)}_{\text{ref}}|^2$ or $|\chi^{(2)}_{\text{scattering}}/\chi^{(2)}_{\text{ref}}|^2$ remain. As the nonlinear susceptibility $\chi^{(n)}_{\text{ref}}$ from the nonlinear crystal remains constant during the measurement, the measured ratio is proportional to the amplitude square of the effective nonlinear susceptibility of the tip and sample $|\chi^{(n)}_{\text{scattering}}|^2$.

In the subsequent processing of measured signals, the anharmonic dependence of the amplitude square of the effective nonlinear susceptibility of the tip/sample $|\chi^{(n)}_{\text{scattering}}|^2$ on the tip mechanical oscillation phase are used to recover the near-field contribution of s-SNOM due to the near-field interaction. The treatment is identical to the procedures described in the main manuscript, with the directly-measured scattering signal replaced by $|\chi^{(n)}_{\text{scattering}}/\chi^{(n)}_{\text{ref}}|^2$ to obtain a waveform of $|\chi^{(n)}_{\text{scattering}}/\chi^{(n)}_{\text{ref}}|^2$ v.s. Φ The near-field contribution extracted in the fashion should have high spatial resolutions, as its cause is the drastic increase of the electric field that is localized at the tip apex. Such field localizations lead to sharp increases in the effective nonlinear susceptibility through the drastic increase of the local electric field, which is also laterally localized under the metallic tip. It allows high spatial resolutions of the method.

Supplementary References

1. Xu, X. G., Gilburd, L., & Walker, G. C. Phase stabilized homodyne of infrared scattering type scanning near-field optical microscopy. *Appl. Phys. Lett.* **105**, 263104 (2014).
